# Genetic analysis of stilbenoid profiles in grapevine stems reveals a major mQTL hotspot on chromosome 18 associated with disease-resistance motifs

**DOI:** 10.1038/s41438-019-0203-x

**Published:** 2019-11-08

**Authors:** Soon L. Teh, Bety Rostandy, Mani Awale, James J. Luby, Anne Fennell, Adrian D. Hegeman

**Affiliations:** 1 Department of Horticultural Science, University of Minnesota, Saint Paul, MN 55108 USA; 2 Agronomy, Horticulture and Plant Science Department, South Dakota State University, Brookings, SD 57007 USA; 3 Present Address: Tree Fruit Research and Extension Center, Department of Horticulture, Washington State University, Wenatchee, WA 98801 USA; 4 Present Address: Department of Mathematics and Statistics, University of North Carolina, Greensboro, NC 27412 USA; 5 Present Address: Grape and Wine Institute, University of Missouri, Columbia, MO 65211 USA

**Keywords:** Plant breeding, Secondary metabolism, Metabolomics

## Abstract

Grapevine (*Vitis* spp.) contains a wealth of phytochemicals that have received considerable attention due to health-promoting properties and biological activities as phytoalexins. To date, the genetic basis of the quantitative variations for these potentially beneficial compounds has been limited. Here, metabolic quantitative trait locus (mQTL) mapping was conducted using grapevine stems of a segregating F_2_ population. Metabolic profiling of grapevine stems was performed using liquid chromatography–high-resolution mass spectrometry (LC-HRMS), resulting in the detection of 1317 ions/features. In total, 19 of these features matched with literature-reported stilbenoid masses and were genetically mapped using a 1449-SNP linkage map and R/qtl software, resulting in the identification of four mQTLs. Two large-effect mQTLs that corresponded to a stilbenoid dimer and a trimer were mapped on chromosome 18, accounting for phenotypic variances of 29.0% and 38.4%. Functional annotations of these large-effect mQTLs on the VitisNet network database revealed a major hotspot of disease-resistance motifs on chromosome 18. This 2.8-Mbp region contains 48 genes with R-gene motifs, including variants of TIR, NBS, and LRR, that might potentially confer resistance to powdery mildew, downy mildew, or other pathogens. The locus also encompasses genes associated with flavonoid and biosynthetic pathways that are likely involved in the production of secondary metabolites, including phytoalexins. In addition, haplotype dosage effects of the five mQTLs further characterized the genomic regions for differential production of stilbenoids that can be applied in resistance breeding through manipulation of stilbenoid production in planta.

## Introduction

Grapes have been recognized as a rich source of phytochemicals, such as phenolic compounds, which are beneficial to plant and human health. A single glass of red wine that is obtained from the fermented extract of 100 to 140 berries contains up to 500 mg of polyphenolic compounds, depending on varieties and vinification methods^1,2^. Within the diversity of polyphenolics, stilbenoids represent a relatively restricted group of phenols that is derived from the general phenylpropanoid pathway, and are found in *Vitaceae* as well as several other plant families^3^. Stilbenoids have received considerable attention due to (1) epidemiological studies attributing moderate consumption of red wine to health benefits, and (2) biological activities as phytoalexins^4–10^.

The production of stilbenoids can be induced by abiotic (e.g., UV irradiation, mechanical injury), as well as biotic (e.g., fungal pathogens) stresses^8–12^. Hypothesized to protect against pathogen infection, stilbenoid production varies across different plant tissues, in terms of concentrations and types. For instance, resveratrol is induced in grape leaves and berries, but constitutive expression and accumulation of resveratrol and other stilbenoids, which are hypothesized to protect against fungal infection, take place primarily in stems and roots^3^.

Due to these activities associated with stilbenoids, there has been significant interest in developing the means to artificially manipulate stilbenoid production in planta^3,13^. Although breeding objectives in grapevine vary by region and market targets, the overarching goal of many programs is to combine high-quality fruit traits with improved biotic (e.g., diseases and pests) and abiotic resistance (e.g., climate and environmental adaptation).

The availability of myriad genetic resources has enabled routine application of genetic markers for parent and seedling selection, or marker-assisted breeding. In addition to genomic resources, metabolomics is a field that is receiving more attention in the area of crop breeding. Metabolomics is an analytical field that provides a comprehensive investigation of metabolite variations in a biological system^14,15^. Coupled with resources from genomics and transcriptomics, metabolomics is developing as an integrative functional tool for crop breeding^16^.

In a classical QTL-mapping experiment, two parents are crossed so that the measurable trait of interest segregates in the offspring family and can be statistically associated with the underlying genetic markers to explain the genetic basis of variation for the trait^17^. Despite the advancement and higher-throughput of modern analytical tools, crop breeding and genetics have continued to rely on traditional phenotyping data. There have been limited reports of chemical profiles being used as metabolic traits for mapping experiments using known metabolic targets associated with flavor and aroma^18–21^, amino acid metabolism^22^, or fruit color^23^, or an untargeted strategy for associating leaf metabolites with complex traits^24^, or with insect resistance^25^.

The aim of this study is to elucidate the genetic basis of stilbenoid variability in grapevine stem tissue through genetic mapping of putative stilbenoid compounds based on accurate masses. A facile application of liquid chromatography–high-resolution mass spectrometry (LC-HRMS)-based metabolic profiling was carried out to identify metabolic QTLs in a segregating experimental population that targets stilbenoids by using an analysis strategy that has been shown to be broadly effective for this compound class^3^, and by quantifying features with masses consistent with previously characterized *Vitis* stilbenoids without a priori knowledge of the specific stilbenoids present in this population.

## Results

### Profiling of F_2_ mapping family showed metabolic inheritance and segregation

**Fig. 1:**
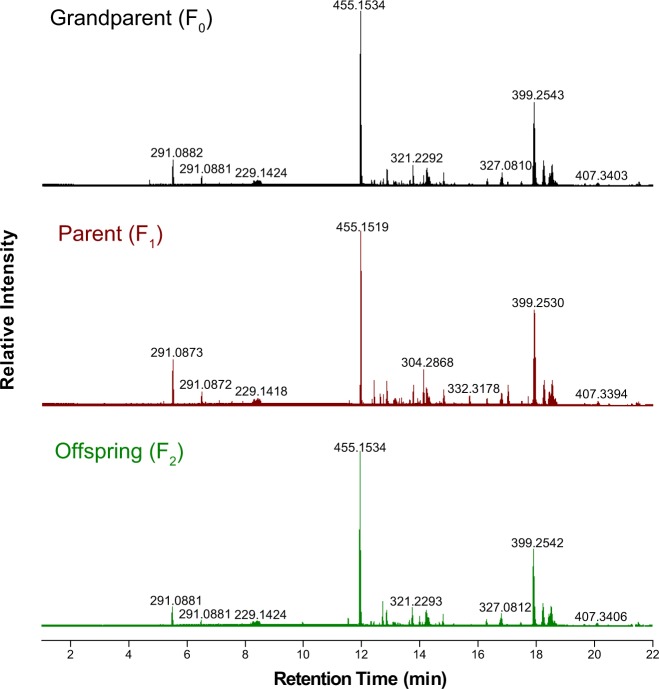
The total ion chromatograms for grape stem extracts of grandparent (F_0_**:** *V. riparia* (USDA PI 588259)), parent (F_1_: *V*. hybrid (16-9-2)), and a representative F_2_ offspring that were analyzed by LC–HRMS

**Fig. 2:**
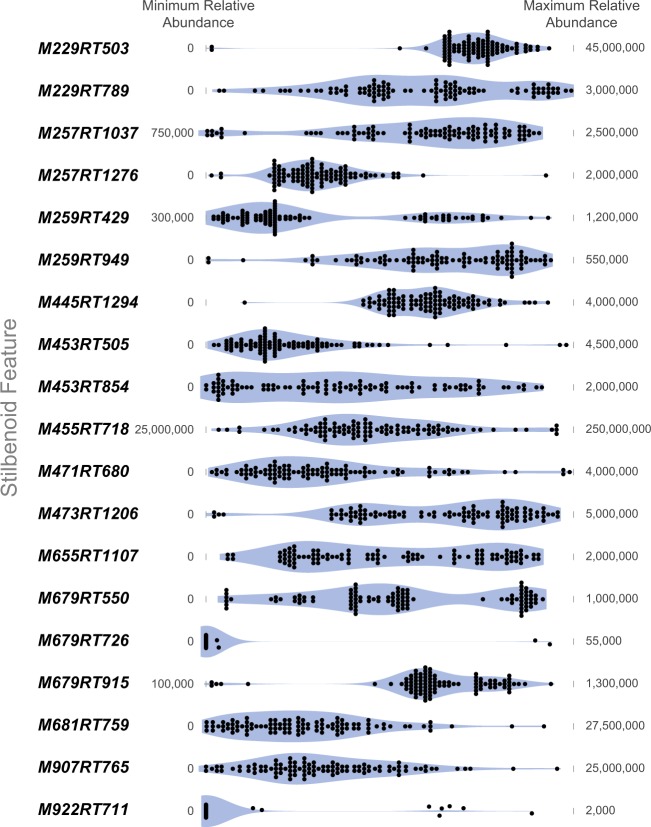
Relative abundance distributions for the 19 identified stilbenoid features

Metabolic profiling of the grapevine stems using LC-HRMS yielded 1317 ions (unique retention time–*m/z* ion pairings) in the [M + H]^+^ mode. An inheritance pattern of chemical profiles was observed when comparing total ion chromatograms (TICs) of female grandparent (F_0_: *V. riparia* (USDA PI 588259)), the parent (F_1_: *V*. hybrid (16-9-2)), and a representative F_2_ progeny (Fig. 1). In addition, segregation among the F_2_ progeny was exhibited upon examination of stilbenoid feature distributions (Fig. 2). The metabolic segregation of ions provided the foundation for mQTL mapping by treating each ion as a metabolic trait.

### Stilbenoid mQTL mapping

**Fig. 3:**
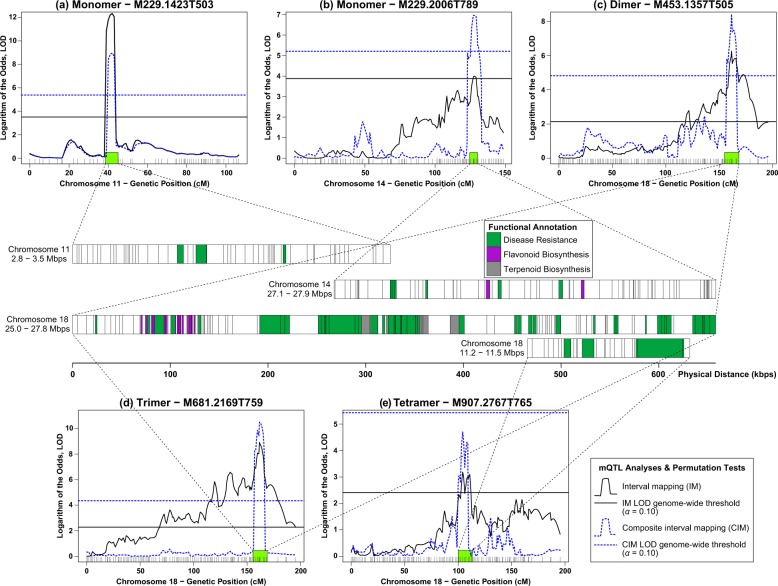
Identification of mQTLs on chromosomes 11, 14, and 18 for five features corresponding to different patterns of stilbenoid oligomerization. **a** monomer–M229.1423T503, **b** monomer– M229.2006T789, **c** dimer– M453.1357T505, **d** trimer–M681.2169T759, and **e** tetramer–M907.2767T765. The GBS marker physical positions relative to the *V. vinifera* reference genome were used to extract genes associated with the mQTL peak. The green, purple, and gray boxes correspond to motifs associated with disease resistance, flavonoid biosynthesis, and terpenoid biosynthesis, respectively. The blue dotted line indicates genome-wide LOD significance thresholds (*α* = 0.10) calculated using 1000 permutations

From the list of 1317 ions, these ions were matched with a library of literature-reported stilbenoid masses (Supplementary Table 1). In total, 19 features matched the library and 17 had sufficient data quality to be used subsequently as metabolic “traits” for mQTL mapping. From 19 features, 4 mQTLs were found for 5 metabolic features (Fig. 3; Table 1) using both interval mapping (IM) and composite interval mapping (CIM) analyses. These features belong to different types of stilbene oligomers. An mQTL for feature M229.1423T503 (*m/z*: 229.1423; retention time: 503 s), a monomeric stilbenoid was mapped on chromosome 11 (2.8–3.5 Mbps) with logarithm of the odds (LOD) scores of 12.33 (IM) and 8.96 (CIM) explaining 4.3% of phenotypic variance. Another monomeric stilbenoid was mapped on chromosome 14 (27.1–27.9 Mbps) with LODs of 3.87 (IM) and 6.96 (CIM), explaining 3.4% of phenotypic variance. Marker–trait association for stilbenoid dimer (M453.1357T505) and trimer (M681.2169T759) identified the same mQTL on chromosomes 18 (25.0–27.8 Mbps) with LODs of 6.26 and 7.69 (IM analysis), explaining 29.0% and 38.4% of phenotypic variances, respectively. In addition, genetic analysis of a tetramer stilbenoid, M907.2767T765 detected an mQTL on chromosome 18 (11.2–11.5 Mbps) with LODs of 3.32 (IM) and 4.70 (CIM) and phenotypic variance of 7.8% (Fig. 3; Table 1).

**Table 1:** Summary of mQTL analysis in the *V. riparia* × “Seyval” F_2_ segregating population

Metabolic feature	Oligomer type	Chromosome	Peak LOD^a^ (Threshold)^b^		Peak position (cM)	LOD interval (cM)^e^	Physical position of LOD interval (Mbps)	Flanking markers	% Phenotypic variance explained
			IM^c^	CIM^d^					
M229.1423T503	Monomer	11	12.33 (3.51)	8.96 (5.38)	42	40–43	2.8–3.5	S11_2824566 S11_3485916	4.3
M229.2006T789	Monomer	14	3.87 (3.77)	6.96 (5.21)	128	126–130	27.1–27.9	S14_27418355 S14_27650708	3.4
M453.1357T505	Dimer	18	6.26 (2.14)	8.02 (4.82)	162	157–166	25.0–27.8	S18_26379100 S18_26392343	29.0
M681.2169T759	Trimer	18	7.69 (2.28)	8.94 (4.35)	162	157–166	25.0–27.8	S18_26379100 S18_26392343	38.4
M907.2767T765	Tetramer	18	3.32 (2.41)	4.70 (5.43)	111	103–112	11.2–11.5	S18_10887948 S18_11730982	7.8

^a^Position of likelihood peak (highest LOD score); logarithm of the odds

^b^Genome-wide LOD thresholds (*α* = 0.10) based on 1000 permutations

^c^Interval mapping

^d^Composite interval mapping

^e^1.8–LOD support interval

### Haplotypic dosage effects of mQTLs

**Fig. 4:**
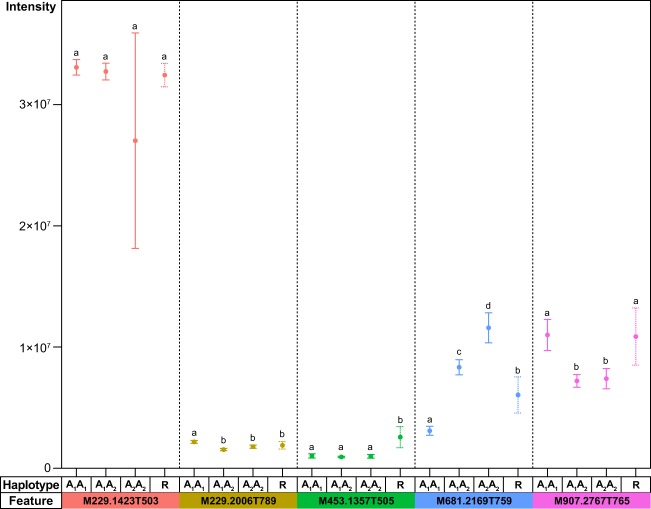
Haplotypic dosage effects of mQTLs. Haplotypes were denoted in a dosage-dependent manner– A_1_A_1_, A_1_A_2_, and A_2_A_2_. Recombinant haplotype was designated as “R”. Letter assignment indicates statistical significance based on Tukey’s significant difference test (*p* < 0.05)

Construction of dosage-dependent haplotypes at each locus enabled the characterization of additive and dominance effects that were associated with differential levels of stilbenoid production. Haplotypes for M229.1423T503 exhibited no statistical difference regardless of allelic dosage. In contrast, haplotypes for the remaining features exhibited dosage-dependent effects. For mQTLs of M229.2006T789 and M907.2767T765, the allelic effects between the homozygotes (i.e., A_1_A_1_ versus A_2_A_2_) were statistically significant and complete dominance was exhibited, where the effect of heterozygote (A_1_A_2_) equaled the effect of homozygous dominant (A_2_A_2_). Meanwhile, haplotypes with partial dominance were observed for mQTL of M681.2169T759 where the effect of A_2_A_2_ > A_1_A_2_ > A_1_A_1_ (Fig. 4).

### An mQTL hotspot on chromosome 18 for disease-resistance genes/motifs

Subsequent to the discovery of mQTLs, the VitisNet database was used to provide functional annotations at the corresponding loci where mQTLs were detected. For the mQTL of feature M229.1423T503, the physical positions span a 0.7-Mbp region on chromosome 11 with 57 annotated genes (54 unique). Three of the genes have roles in disease resistance and plant response to biotic stresses. The mQTL of M229.2006T789 spans a 0.8-Mbp region on chromosome 14 with 74 functionally annotated genes, of which six genes were involved in plant–pathogen interaction, including disease-resistance genes. Meanwhile, the mQTL for feature M907.2767T765 was localized to a 0.3-Mbp region on chromosome 18 with 27 annotated genes. Of these, five genes were annotated as having possible roles in disease resistance and plant biotic stresses (Fig. 3; Table 2; Supplementary File 1).

**Table 2:** Functional annotations for mQTLs

Metabolic feature (oligomer type)	Beginning marker	End marker	Gene ID (VIT_xxx)^a^	VitisNet pathway ID^b^
M229.1423T503 (monomer)	chr11_02825398_02830030	chr11_03485684_03493115	11s0016g03460 to 11s0016g04210	vv10062, vv10195, vv10252, vv11000, vv23008, vv23020, vv23060, vv24120, vv30003, vv30008, *vv34627*, vv40006, vv44110, vv44145, *vv44810*, vv50110, vv50121, *vv50122*, *vv50124*, *vv60022*, *vv60037*, vv60062, vv60080
M229.2006T789 (monomer)	chr14_27118108_27119063	chr14_27924886_27928982	14s0066g00620 to 14s0066g01550	vv10010, vv10020, vv10052, vv10053, vv10061, vv10190, vv10252, vv10400, vv10511, vv10530, *vv10860*, *vv10908*, vv10910, vv10940, *vv11002*, vv23010, vv23040, vv30009, vv34020, vv40006, vv44110, *vv44810*, vv50110, vv50121, *vv50122*, vv50131, vv60007, vv60018, vv60034, vv60044
M453.1357T505 (dimer)	chr18_25040622_25045542	chr18_27779526_27785425	18s0041g00580 to 18s0041g02470; 18s0089g00010 to 18s0089g00090	vv10230, vv10563, *vv10592*, vv10630, vv10770, *vv10860*, vv10902, *vv10904*, *vv10941*, *vv10942*, *vv11002*, vv23015, *vv23030*, vv23040, *vv34627*, vv40006, *vv44810*, *vv50122*, *vv50123*, vv50133, vv52010, vv60011, *vv60040*, vv60042, vv60046, vv60082
M681.2169T759 (trimer)	chr18_25040622_25045542	chr18_27779526_27785425	18s0041g00580 to 18s0041g02470; 18s0089g00010 to 18s0089g00090	vv10230, vv10563, *vv10592*, vv10630, vv10770, *vv10860*, vv10902, *vv10904*, *vv10941*, *vv10942*, *vv11002*, vv23015, *vv23030*, vv23040, *vv34627*, vv40006, *vv44810*, *vv50122*, *vv50123*, vv50133, vv52010, vv60011, *vv60040*, vv60042, vv60046, vv60082
M907.2767T765 (tetramer)	chr18_11201495_11206144	chr18_11496666_11502715	18s0001g13150 to 18s0001g13430	vv10052, vv10190, *vv10740*, *vv10908*, vv23022, vv24120, vv30008, vv50110, *vv50122*, vv60034, *vv60059*

^a^Truncated designation (e.g., VIT_11s0016g03460 was reported as 11s0016g03460)

^b^Pathway IDs with significant enrichment (Fisher’s exact test, 1000 permutations and permuted *p*-value of *p* < 0.05) are underlined and italicized

Summary of metabolic features/traits, physical positions spanning the loci, gene ID and VitisNet pathway ID

A major-effect locus explaining 29.0% and 38.4% phenotypic variance was co-localized for M453.1357T505 and M681.2169T759, respectively. The mapped physical positions span a 2.7-Mbp on chromosome 18 with 147 functionally annotated genes (Fig. 3). A significant enrichment was identified for the R proteins from plant–pathogen interaction (vv34627) and the diterpenoid biosynthesis (vv10904) VitisNet pathways, with 13 and 5 genes annotated to the pathway (Table 2; Supplementary File 1). A large number of additional genes not annotated to the pathway but with identified roles in disease resistance were also found in the locus, predominantly Toll/interleukin-1 receptor (TIR)–nucleotide-binding site (NBS)–leucine-rich repeat (LRR) (Supplementary File 1). In addition, 15 genes were annotated as being involved in flavonoid biosynthetic pathways, as were six genes in terpenoid biosynthesis with putative function in hydroxylation.

## Discussion

### Mapping loci using variation in metabolite levels of the progeny

Genes that are involved in a biosynthesis pathway have been identified either through forward genetics (i.e., screening the phenotype to identify gene(s) responsible for a trait of interest) or reverse genetics (i.e., evaluating the phenotype subsequent to disruption of gene function). In traditional crop breeding, the former case has been routinely applied, resulting in QTL discovery, fine mapping, candidate gene cloning, and finally, functional identification. Despite the eventual identification of genes, it remains unclear which of the genes account for the variation in metabolite levels across tissue types and genotypes. Alternatively, genetic mapping of metabolites provides a solution to elucidate putative genes that underlie metabolic variations.

In this study, an integrated approach in an F_2_ family of 101 offspring linking a genetic map of 1449 SNPs and a metabolomics data set of 1317 metabolic features that was coupled with a library of stilbenoid masses resulted in the discovery of five mQTLs. In addition, the use of a high-resolution mass spectrometer provided accurate mass detection of ions from a complex chemical extract, thereby enabling high-throughput analysis of the mapping family. Without a priori knowledge of compound identity, metabolic profiling of an experimental population can provide thousands of metabolic features to be evaluated in mQTL mapping. Similar to the conventional marker–trait association where marker information is derived from a genetic linkage map while trait information is acquired from a variation for phenotype of the trait, a metabolic feature can be treated as a “trait” to assess marker–“trait” (feature) association^22,24^. Significant variation in ion abundance/intensity is apparent across the 19 features (Fig. 2). Since the observed distributions of features are multimodal for segregating traits, and can often be confused with skewed single"-modal distributions, we opted to perform the QTL analysis without transformation. Although not required for our data set, log_2_ and log_10_ transformations can be performed to normalize the distribution for other analyses, as needed.

### Matching features with a library of stilbenoid masses prior to mQTL mapping

From the list of 1317 metabolic features, the vast majority of features did not match with a library of stilbenoid masses that have been reported in the literature (Supplementary Table 1). This guided approach enabled a targeted list of 19 features to be examined for mQTL mapping. Subsequent mQTL analysis (with genome-wide threshold of *α* = 0.10) identified four mQTLs. Despite the suitability of this method, there are several reasons why the number of mQTLs may have been underestimated (i.e., false negatives). First, complex genetic regulation of metabolites in a pathway could hamper the detection of mQTLs. The production of one metabolite is likely controlled by several small-effect loci, which may not be detected during the mapping analysis. In addition, the complex genetic backgrounds of an already highly heterozygous crop may help explain the presence of small-effect loci, rendering a lower-resolution mQTL mapping. The ancestry of the F_2_ mapping family used for this experiment is comprised of at least six *Vitis* species, including *V. riparia*, *V. vinifera*, *V. rupestris*, *V. labrusca*, *V. aestivalis*, and *V. berlandieri*. Relevant marker loci of the highly interspecific hybrid progeny can be lost due to anchoring on the *V. vinifera* reference genome during the GBS process. Finally, the ion signal of metabolic features could be above the noise threshold for some offspring, but within the noise threshold for other offspring, likely resulting in no mQTL detection.

### mQTLs on chromosome 18 are coincident with disease-resistance genes/motifs

In a 2.7-Mbp region on chromosome 18, a hotspot of disease-resistance genes was jointly detected for features M453.1357T505 and M681.2169T759 (Fig. 3; Table 1). The lack of genetic resolution is likely due to the reduced recombination events in this relatively small F_2_ family. Although the original F_2_ family is comprised 424 progeny, dormant cane sections were acquired from only a subset of this family (i.e., *N* = 101)^26^. A comprehensive analysis using the entire family would likely resolve the mQTLs detected for features M453.1357T505 and M681.2169T759.

From the network database, a third of known genes (Chr. 18: 25.0–27.8 Mbps) were saturated with disease-resistance motifs, such as TIR-NBS-LRR, TIR-NBS, LRR family protein, and R protein (Table 2; Supplementary File 1). They were annotated as “R proteins from plant–pathogen interaction” and fell into categories of “biotic stress response”, “response to stimuli”, “plant–pathogen interaction”, as well as “transposable elements”. Along with the statistical reports of 29.0% and 38.4% phenotypic variance, this genomic region is a major-effect locus for disease resistance, indicating a major potential hotspot for the biosynthesis of defense metabolites.

The detection of a large-effect disease-resistance hotspot coincident with mQTLs for stilbenoid dimers and trimmers, but not for monomers, is consistent with previous findings that the type, rather than the amount of stilbenoids are more important in grapevine resistance to pathogens^3,9,10,27,28^. Though previously touted as an important phytoalexin in plant defense, resveratrol (i.e., a stilbenoid monomer) has been reported to be less fungitoxic than stilbenoid dimers and trimers, such as viniferins^9,10^.

In a recent QTL mapping of downy mildew and stilbenoid induction, Vezzulli et al. reported a list of candidate genes underlying QTLs for downy mildew severity and incidence, also mapped on chromosome 18^29^. These candidate genes overlap with our major hotspot of disease-resistance motifs on chromosome 18. In addition in Vezzulli et al.’s findings, most polyphenol-related QTLs were mapped on chromosome 16, while only *cis*- and *trans*-piceid, astringin, isorhapontin, *cis*-*Ɛ*-viniferin, and the sum of monomeric stilbenoid abundance were mapped on chromosome 18. Stilbenoid mQTLs identified in this study were found on chromosome 18, overlapping the down mildew resistance region described by Vezzulli et al.^29^.

Coincidentally, the identification of a disease-resistance hotspot on chromosome 18 co-localizes with previous QTL reports for downy mildew and powdery mildew resistance in grapevine. Di Gaspero et al. mapped the *Rpv3* (resistance to *Plasmopara viticola* or downy mildew) locus on chromosome 18 and reported haplotypic structure at six microsatellite loci spanning a 1.2-Mbp region from 24.8 to 26.0 Mbp on chromosome 18, a region overlapping with our reported mQTLs^30^. The *Rpv3* locus was also linked with stilbenoid induction in a recent interspecific grapevine population mapping study conducted by Vezzulli et al.^29^. The resistance locus was inherited from “Seyval”, which is a grandparent in our F_2_ mapping family. Another report on grapevine downy mildew resistance also detected a major locus on chromosome 18, being strongly associated with GF18-06 and GF18-08 markers that were mapped on the 25.9–26.9-Mbp region^31^. The resistant parent of the experimental population was GF.GA-47-42, which was a cross between “Bacchus” and “Seyval”^31^. Meanwhile, in grapevine powdery mildew-resistance mapping, the *Run2* (resistance to *Uncinula necator*) was mapped on chromosome 18 for four traits—leaf, cane, rachis, and fruit^32^. The locus is closely linked to the VMC7f2 marker, anchored at a physical position of 26.9 Mbp^32^, which is located within the 25.0–27.8-Mbp region of disease-resistance motifs.

Taken together, the co-localization of our mQTLs on chromosome 18 with a major disease-resistance hotspot, and three QTL findings on grapevine downy and powdery mildews implied further support that the region is likely associated with downy and powdery mildew resistance. In the case of downy mildew resistance, the resistance donor parent or progenitor in the experimental crosses is “Seyval”, the same F_0_ grandparent in our experimental F_2_ segregating family^30,31^. Given the preliminary observations of our analysis, further experiments need to be carried out to characterize the roles of this region in conferring resistance.

In addition to disease-resistance motifs, genes of two other annotated metabolic pathways were quite ubiquitous in this region (Chr. 18: 25.0–27.8 Mbps). Genes involved in flavonoid and terpenoid biosynthetic pathways (Fig. 3), that are involved in the production of various secondary metabolites, including those involved in plant defense response, were associated with the loci^33,34^.

### mQTLs on chromosomes 11, 14, and 18 associated mainly with primary metabolism

With the exclusion of a handful of disease-resistance genes, the genomic regions for mQTLs associated with features M229.1423T503, M229.2006T789, and M907.2767T765 appear to be populated with a suite of genes linked with primary metabolism that are not obviously connected to stilbenoid metabolism. In combination with the observation of disease-resistance motifs highlighted in the aforementioned mQTL hotspot, it is likely that these regions may also be associated with plant–pathogen interaction. Secondary metabolism is of great interest in plant–pathogen interaction because phytopathogen infection induces a plant’s defense program. However, less is known about the effects of pathogenic attack on primary metabolism. This is especially important because most attacks (e.g., parasitic relationship) result in yield losses without killing the crops. In particular, various aspects of photosynthesis, assimilate partitioning, and source–sink relationship are downstream physiological changes of the infected tissues that need to be investigated to understand the mechanisms and consequences of a plant–pathogen interaction^35–37^.

Based on the network analysis, some of the functional annotations in these genomic windows include the signaling pathway, macromolecule transport, transcription regulation, ubiquitin-mediated proteolysis, nucleic acid metabolism, carbohydrate metabolism, and glycosyl transference (Supplementary File 1)^38^. In addition to genes related to primary metabolism, the annotation indicates a peroxidase gene that appears to be involved in the metabolism of phenylalanine, a precursor to various biosynthetic pathways, such as phenylpropanoid, flavonoid, and stilbenoid pathways. Similar peroxidases have been implicated in stilbenoid oligomerization and so may be directly involved in the coupling of higher-order stilbenoids^13^. Taken together, it is likely that these three mQTL regions may be linked with plant–pathogen interaction, both in primary and secondary metabolism.

## Conclusion

In this study, we demonstrated the utility of combining analytical tools (i.e., metabolic profiling) and large genomic data sets to characterize the genetic basis of metabolites. Despite the absence of compound identity and structures, the use of high-resolution mass spectrometer provided detection of various ions that can be treated as metabolic “traits” and coupled with genetic maps for mQTL mapping. Understanding the genetic controls of potentially bioactive compounds (e.g., stilbenoids) can assist breeders and viticulturists to select genotypes (e.g., seedlings, parents, and existing cultivars) with increased levels of these biomarkers through marker-assisted breeding.

## Materials and methods

### Sampling of plant materials

A segregating F_2_ mapping family was derived from self-pollination of a single hermaphrodite F_1_ individual (16-9-2), which was a hybrid from the initial F_0_ cross of *V. riparia* 37 (USDA PI 588259) × “Seyval” (Seyve-Villard 5-276), as previously described by Fennell et al.^39^. The experimental family was grown and maintained in the field in Brookings, SD. The original F_2_ mapping family consists of 424 offspring that has been mapped for QTLs of enological and environmental adaptation traits^26,40^. In this study, a subset (*N* = 101) from the pilot-mapping family was used for metabolic profiling and mQTL mapping. Two to four cuttings of approximately three to five inches of dormant woody stems from each of 101 progeny were collected between February 26, 2014 and March 12, 2014. Samples were wrapped in aluminum foil to prevent moisture loss, shipped on dry ice to Minnesota, and stored at –80 °C until extraction and analysis.

### Sample preparation and extraction

Woody stems were lyophilized for 24 to 36 h until all moisture was removed. For each offspring, the dried stems were ground together with a conventional coffee grinder (Kuissential™ Ceramic Burr Coffee Grinder). In-between samples, the grinder was cleaned and air-dried. The ensuing steps, including chemical extraction, sonication, centrifugation, evaporation, and adjusted reconstitution, were performed based on a facile extraction method that was described by Pawlus et al.^27^.

### Metabolic profiling with LC-HRMS

An aliquot (1 μL) of each reconstituted extract (0.2 μg/μL) was injected and analyzed on an UltiMate 3000 UHPLC coupled to a Q Exactive Hybrid Quadruple-Orbitrap mass spectrometer (Thermo Fisher Scientific, USA). Samples were chromatographically resolved at a flow rate of 0.45 mL/min on a C_18_-reverse-phase column (HSS T3, 2.1 mm i.d. ×100 mm, 1.8-μm particle size; Waters, Milford, MA) by mixing mobile-phase solvent A (water with 0.1% formic acid) and solvent B (acetonitrile with 0.1% formic acid) to generate the following gradient: 0 to 1 min, 2% B; 1 to 10 min, 2 to 30% B; 10 to 12 min, 30 to 50% B; 12 to 20 min, 50 to 75% B; 20 to 22 min, 75 to 98% B; 22 to 23 min, 98 to 2% B; 23 to 27 min, 2% B. The mass spectrometer was operated in the positive/negative switching ionization mode over a scan range of 150–2000 *m/z*. The presence of monomers, dimers, trimers, and tetramers was monitored using the [M + H]^+^ *m/z* values of 229, 445, 681, and 907, respectively. Ions yielded in the [M–H]^−^ mode were not used for mQTL mapping due to ionization issues. UV/visible absorbance data were simultaneously collected using the UltiMate 3000 UHPLC diode array detector throughout each separation; spectra with *λ*_max_ values of 280 nm and 306 nm were consistent with eluting stilbenoids.

### Data pre-processing

Metabolomics data from LC-HRMS were processed in XCMS for peak alignment with parameter settings optimized for the analysis (method = centWave; peakwidth = 5,20; snthresh = 10; ppm = 3.0; mzdiff = 0.01)^41^. The aligned peaks were then subjected to grouping, retention time correction, and regrouping with optimized parameters (bw = 2; mzwid = 0.015; minfrac = 0.1). Following feature grouping and correction, peak filling was performed using chromatography method in XCMS, allowing integration of the area under the curve of samples that might have been missed during the group step.

However, XCMS peak picking software might still miss the reporting of features in samples with lower abundance or absence of metabolite ions, resulting in zero entries in the generated peak table. These zero entries are considered missing values. In this experiment, imputation of missing values was not performed, but features with > 10% missing values in the offspring samples were not considered for subsequent QTL analysis to avoid potential erroneous QTL detection. Of the 19 stilbenoid features, 14 contain no zero-intensity values, 1 (M453RT854) contains a single zero-intensity value, 2 (M681RT759 & M907RT765) contain 2 zero-intensity values, and 2 (M922T711 and M679T726) were ultimately excluded with 93 zero-intensity values, and 97 zero-intensity values respectively.

### Linking metabolic features with a library of stilbenoid masses

A list of accurate masses corresponding to 54 structurally characterized stilbenoids was compiled from the literature (Supplementary Table 1). Using this list, 19 features, out of the 1317 extracted using XCMS, were found to match stilbenoid masses from this list. All 19 features were confirmed manually by examining extracted ion chromatograms (EICs) generated from the raw data files using Xcalibur 4.0 data visualization software for each corresponding accurate mass value.

### Stilbenoid metabolic quantitative trait locus (mQTL) mapping

The intensities for each feature (with a mass matching a stilbenoid and a unique retention time) were compiled for each individual for subsequent mQTL mapping; each individual stilbenoid metabolic feature (unique mass/retention time pair) was treated as a metabolic “trait” for the analysis.

A previously described genotype-by-sequencing single-nucleotide polymorphism (SNP) map with 1449 markers over 19 chromosomes was used for QTL analysis of 17 (2 were excluded with >10% missing values) of the 19 identified stilbenoid features^26^. As described by Yang et al., the F_2_ genetic map is comprised < hkxhk > markers with linkages estimated using cross-pollination cross type^26^. QTLs with a recessive allele that cannot be detected in F1 may be detected in F2 because of the change in segregation type from < lmxll > or < nnxnp > (2 genotypic classes) to < hkxhk > (three genotypic classes). Targeted mQTL detection was conducted on R/qtl software (version 3.5.3) using interval mapping (IM) and composite interval mapping (CIM)^42^. Interval mapping was performed using scanone function (R/qtl) with expectation–maximization (EM) algorithm. Composite interval mapping was conducted using cim() function with the Kosambi map function. The minimum logarithm of odds (LOD) score for mQTL detection was determined by genome-wide LOD significance thresholds (*α* = 0.10) calculated using 1000 permutations.

### Haplotype construction and analysis of mQTLs

A haplotype is defined by a combination or a group of alleles that tend to be inherited together. Haplotype was constructed by assigning alleles to offspring in a family based on observed marker alleles that had already been assigned during QTL analysis. At each mQTL, functional SNP haplotype spanning the locus was constructed for the progeny based on differential intensities of the feature. Given that < hk × hk > marker type was used in the SNP map, mQTL haplotyping was reported as haplotypic dosage effects (i.e., A_1_A_1_, A_1_A_2_, and A_2_A_2_). A recombinant haplotype was designated where there was a recombination at the mQTL region. Analysis of variance (ANOVA) was performed to determine if there was a statistical difference owing to these haplotype dosage effects. A post hoc Tukey’s analysis was used to identify significant difference (*p* < 0.05).

### Functional annotation of mQTLs

To gain more insights into the mQTL regions, the physical positions of the markers defining the mQTLs were used to identify genes associated with the loci using the PN40024 12 × .v2, V2 annotation^38,43^. Gene functional annotation and VitisNet pathways were used to explore genes underlying the loci of interest. Genes associated with the QTL were tested for enrichment in VitisNet Pathways, using a 1000 permutations, a Fisher’s exact test of *p* < 0.05 and a permuted *p*-value of *p* < 0.05^38,44^.

## Supplementary information

Supplementary Table 1

Supplementary File 1
