# Supplementary material for: Genetic analysis of stilbenoid profiles in grapevine stems reveals a major mQTL hotspot on chromosome 18 associated with disease-resistance motifs
Source: Hortic Res. 2019 Nov 8;6:121. doi: 10.1038/s41438-019-0203-x (PMC6838171; doi:10.1038/s41438-019-0203-x)
Supplement: Supplementary file 1 — Supplementary Table 1 [file 41438_2019_203_MOESM1_ESM.docx]

# **Genetic Analysis of Stilbene Profiles in Grapevine Stems Reveals a Major mQTL Hotspot on Chromosome 18 Associated with Disease Resistance Motifs**

## **Horticulture Research**

### **Supplementary Table 1**

Soon Li Teh^1,†^, Bety Rostandy^1,††^, Mani Awale^2,‡^, James J. Luby^1^, Anne Fennell^2^, Adrian D. Hegeman^1,^*

^1^ Department of Horticultural Science, University of Minnesota, Saint Paul, MN 55108, USA

^2^ Agronomy, Horticulture and Plant Science Department, South Dakota State University, Brookings, SD 57007, USA

Present Addresses:

^†^ Tree Fruit Research and Extension Center, Department of Horticulture, Washington State University, Wenatchee, WA 98801, USA

^††^ Department of Mathematics and Statistics, University of North Carolina, Greensboro, NC 27412, USA

^‡^ Grape and Wine Institute, University of Missouri, Columbia, MO 65211, USA

* Correspondence: Adrian D. Hegeman (hegem007@umn.edu)

**Supplementary Table 1.** A compiled list of stilbene oligomer/class, name, chemical formulae, and masses for stilbenes that have been reported in the literature.

| **Oligomer** | **Compound** | **Formula** | **Exact Mass** | **[M+H]^+^** | **References** |
| --- | --- | --- | --- | --- | --- |
| Monomer | Resveratrol | C_14_H_12_O_3_ | 228.0786 | 229.0865 | 17, 26 |
|  | Piceatannol | C_14_H_12_O_4_ | 244.0736 | 245.0814 | 2, 5 |
|  | Pterostilbene | C_16_H_16_O_3_ | 256.1099 | 257.1178 | 21 |
|  | Rhapontigenin | C_15_H_14_O_4_ | 258.0892 | 259.0970 | 1 |
|  | Piceid | C_20_H_22_O_8_ | 390.1315 | 391.1393 | 26 |
|  | Resveratroloside | C_20_H_22_O_8_ | 390.1315 | 391.1393 | 20, 35 |
|  | Astringin | C_20_H_22_O_9_ | 406.1264 | 407.1342 | 20, 35 |
| Dimer | Vitisinol C | C_27_H_24_O_5_ | 428.1624 | 429.1702 | 13 |
|  | Vitisinol E | C_27_H_24_O_6_ | 444.1573 | 445.1651 | 3, 4 |
|  | Viniferifuran | C_28_H_20_O_6_ | 452.1260 | 453.1338 | 9, 16 |
|  | Vitisinol A | C_28_H_20_O_6_ | 452.1260 | 453.1338 | 13 |
|  | Vitisinol G | C_28_H_20_O_6_ | 452.1260 | 453.1338 | 3 |
|  | Ampelopsin D | C_28_H_22_O_6_ | 454.1416 | 455.1495 | 8 |
|  | Viniferin | C_28_H_22_O_6_ | 454.1416 | 455.1495 | 27, 30 |
|  | Vitisinol D | C_28_H_22_O_6_ | 454.1416 | 455.1495 | 13 |
|  | Malibatol A | C_28_H_20_O_7_ | 468.1209 | 469.1287 | 36 |
|  | Ampelopsin A | C_28_H_22_O_7_ | 470.1366 | 471.1444 | 29, 32 |
|  | Scirpusin A | C_28_H_22_O_7_ | 470.1366 | 471.1444 | 18 |
|  | Amurensin A | C_28_H_24_O_7_ | 472.1522 | 473.1600 | 8 |
|  | Vitisinol B | C_35_H_26_O_8_ | 574.1628 | 575.1706 | 13 |
| Trimer | Vitisinol F | C_41_H_34_O_8_ | 654.2254 | 655.2332 | 3 |
|  | Amurensin C | C_42_H_30_O_9_ | 678.1890 | 679.1968 | 11 |
|  | Amurensin D | C_42_H_30_O_9_ | 678.1890 | 679.1968 | 11 |
|  | α-viniferin | C_42_H_30_O_9_ | 678.1890 | 679.1968 | 31 |
|  | Ampelopsin C | C_42_H_32_O_9_ | 680.2046 | 681.2125 | 22 |
|  | Ampelopsin E | C_42_H_32_O_9_ | 680.2046 | 681.2125 | 8, 23 |
|  | Amurensin B | C_42_H_32_O_9_ | 680.2046 | 681.2125 | 7 |
|  | Amurensin G | C_42_H_32_O_9_ | 680.2046 | 681.2125 | 10 |
|  | Miyabenol C | C_42_H_32_O_9_ | 680.2046 | 681.2125 | 27 |
|  | Viniferol D | C_42_H_32_O_9_ | 680.2046 | 681.2125 | 34 |
|  | Vitisin E | C_42_H_32_O_9_ | 680.2046 | 681.2125 | 33 |
| Tetramer | Amurensin I | C_56_H_38_O_12_ | 902.2363 | 903.2442 | 12 |
|  | Amurensin M | C_56_H_38_O_12_ | 902.2363 | 903.2442 | 12 |
|  | Amurensin L | C_56_H_40_O_12_ | 904.2520 | 905.2598 | 12 |
|  | Vitisifuran A | C_56_H_40_O_12_ | 904.2520 | 905.2598 | 12,16 |
|  | Vitisifuran B | C_56_H_40_O_12_ | 904.2520 | 905.2598 | 16 |
|  | Ampelopsin H | C_56_H_42_O_12_ | 906.2676 | 907.2755 | 27 |
|  | Miyabenol A | C_56_H_42_O_12_ | 906.2676 | 907.2755 | 3, 13 |
|  | Amurensin J | C_56_H_42_O_12_ | 906.2676 | 907.2755 | 12 |
|  | Davidol A | C_56_H_42_O_12_ | 906.2676 | 907.2755 | 23 |
|  | Flexuosol A | C_56_H_42_O_12_ | 906.2676 | 907.2755 | 24 |
|  | Heyneanol A | C_56_H_42_O_12_ | 906.2676 | 907.2755 | 22 |
|  | Hopeaphenol | C_56_H_42_O_12_ | 906.2676 | 907.2755 | 15, 32 |
|  | Isohopeaphenol | C_56_H_42_O_12_ | 906.2676 | 907.2755 | 15 |
|  | Vaticanol C | C_56_H_42_O_12_ | 906.2676 | 907.2755 | 27 |
|  | Viniferol A | C_56_H_42_O_12_ | 906.2676 | 907.2755 | 36 |
|  | Viniferol B | C_56_H_42_O_12_ | 906.2676 | 907.2755 | 37 |
|  | Viniferol C | C_56_H_42_O_12_ | 906.2676 | 907.2755 | 37 |
|  | Vitisin A | C_56_H_42_O_12_ | 906.2676 | 907.2755 | 19, 28 |
|  | Vitisin B | C_56_H_42_O_12_ | 906.2676 | 907.2755 | 19, 25 |
|  | Vitisin C | C_56_H_42_O_12_ | 906.2676 | 907.2755 | 14 |
|  | Vitisin D | C_56_H_42_O_12_ | 906.2676 | 907.2755 | 33 |
|  | Amurensin K | C_56_H_40_O_13_ | 920.2469 | 924.2782 | 12 |
|  | Viniferol E | C_56_H_44_O_13_ | 921.2547 | 925.2860 | 6 |
| **References:**   1. Adrian, M., Jeandet, P., Douillet-Breuil, A. C., Tesson, L., & Bessis, R. (2000). Stilbene content of mature Vitis vinifera berries in response to UV-C elicitation. *Journal of Agricultural and Food Chemistry*, *48*(12), 6103-6105. 2. Bavaresco, L., Fregoni, M. A. R. I. O., Trevisan, M. A. R. C. O., Mattivi, F., Vrhovsek, U., & Falchetti, R. (2002). The occurrence of the stilbene piceatannol in grapes. *Vitis*, *41*(3), 133-136. 3. Chiou, W. F., Shen, C. C., Chen, C. C., Lin, C. H., & Huang, Y. L. (2009). Oligostilbenes from the roots of Vitis thunbergii. *Planta medica*, *75*(08), 856-859. 4. Choi, Y. H., Yoo, M. Y., Choi, C. W., Cha, M. R., Yon, G. H., Kwon, D. Y., ... & Ryu, S. Y. (2009). A new specific BACE-1 inhibitor from the stembark extract of Vitis vinifera. *Planta medica*, *75*(05), 537-540. 5. Delaunay, J. C., Castagnino, C., Chèze, C., & Vercauteren, J. (2002). Preparative isolation of polyphenolic compounds from Vitis vinifera by centrifugal partition chromatography. *Journal of chromatography A*, *964*(1-2), 123-128. 6. Fujii, F., He, Y. H., & Terashima, K. (2005). Three New Stilbeneoligomers from the Roots of Vitis vinifera" Kyohou". *Heterocycles*, *65*(10), 2461-2469. 7. Kim, H., Thuong, P. T., Ngoc, T. M., Lee, I., Hung, N. D., & Bae, K. (2009). Antioxidant and lipoxygenase inhibitory activity of oligostilbenes from the leaf and stem of Vitis amurensis. *Journal of ethnopharmacology*, *125*(2), 304-309. 8. Huang, K. S., & Lin, M. (1999). Oligostilbenes from the roots of Vitis amurensis. *Journal of Asian natural products research*, *2*(1), 21-28. 9. Huang, K. S., Mao, L. I. N., & Wang, Y. H. (1999). Synthesis of Amurensin H, a New Resveratrol Dimerfrom the Roots of Vitis Amurensis [J]. *中国化学快报 (英文版)*, *10*. 10. Huang, K. S., Lin, M., Yu, L. N., & Kong, M. (1999). A new oligostilbene from the roots of Vitis amurensis. *Chinese Chemical Letters*, *10*(9), 775-776. 11. Huang, K. S., Lin, M., Yu, L. N., & Kong, M. (2000). Four novel oligostilbenes from the roots of Vitis amurensis. *Tetrahedron*, *56*(10), 1321-1329. 12. Huang, K. S., Lin, M., & Cheng, G. F. (2001). Anti-inflammatory tetramers of resveratrol from the roots of Vitis amurensis and the conformations of the seven-membered ring in some oligostilbenes. *Phytochemistry*, *58*(2), 357-362. 13. Huang, Y. L., Tsai, W. J., Shen, C. C., & Chen, C. C. (2005). Resveratrol Derivatives from the Roots of Vitis t hunbergii. *Journal of natural products*, *68*(2), 217-220. 14. Ito, J., & Niwa, M. (1996). Absolute structures of new hydroxystilbenoids, vitisin C and viniferal, from Vitis vinifera ‘Kyohou’. *Tetrahedron*, *52*(30), 9991-9998. 15. Ito, J., Niwa, M., & Oshima, Y. (1997). A new hydroxystilbene tetramer named isohopeaphenol from Vitis Vinifera Kyohou. *Heterocycles*, *9*(45), 1809-1813. 16. Ito, J., Takaya, Y., Oshima, Y., & Niwa, M. (1999). New oligostilbenes having a benzofuran from Vitis vinifera ‘Kyohou’. *Tetrahedron*, *55*(9), 2529-2544. 17. Jeandet, P., Bessis, R., Maume, B. F., Meunier, P., Peyron, D., & Trollat, P. (1995). Effect of enological practices on the resveratrol isomer content of wine. *Journal of Agricultural and Food Chemistry*, *43*(2), 316-319. 18. Kong, Q., Ren, X., Jiang, L., Pan, Y., & Sun, C. (2010). Scirpusin A, a hydroxystilbene dimer from Xinjiang wine grape, acts as an effective singlet oxygen quencher and DNA damage protector. *Journal of the Science of Food and Agriculture*, *90*(5), 823-828. 19. Korhammer, S., Reniero, F., & Mattivi, F. (1995). An oligostilbene from Vitis roots. *Phytochemistry*, *38*(6), 1501-1504. 20. Krisa, S., Larronde, F., Budzinski, H., Decendit, A., Deffieux, G., & Mérillon, J. M. (1999). Stilbene production by Vitis vinifera cell suspension cultures: methyl jasmonate induction and 13C biolabeling. *Journal of Natural Products*, *62*(12), 1688-1690. 21. Langcake, P., Cornford, C. A., & Pryce, R. J. (1979). Identification of pterostilbene as a phytoalexin from Vitis vinifera leaves. *Phytochemistry*, *18*(6), 1025-1027. 22. Li, W. W., Ding, L. S., Li, B. G., & Chen, Y. Z. (1996). Oligostilbenes from Vitis heyneana. *Phytochemistry, 42*(4), 1163-1165. 23. Li, W. W., Li, B., & Chen, Y. Z. (1998a). Oligostilbenes from Vitis davidii. *Chinese Chem. Lett., 9, 735-736.* 24. Li, W. W., Li, B. G., & Chen, Y. Z. (1998b). Flexuosol A, a new tetrastilbene from Vitis flexuosa. *Journal of natural products*, *61*(5), 646-647. 25. Mattivi, F., & Reniero, F. (1992). Oligostilbenes from the roots of genus Vitis. *Bulletin de Liaison Groupe Polyphenols (France)*. 26. Mattivi, F., Reniero, F., & Korhammer, S. (1995). Isolation, characterization, and evolution in red wine vinification of resveratrol monomers. *Journal of Agricultural and Food Chemistry*, *43*(7), 1820-1823. 27. Mattivi, F., Vrhovsek, U., Malacarne, G., Masuero, D., Zulini, L., Stefanini, M., ... & Guella, G. (2011). Profiling of resveratrol oligomers, important stress metabolites, accumulating in the leaves of hybrid Vitis vinifera (Merzling× Teroldego) genotypes infected with Plasmopara viticola. *Journal of agricultural and food chemistry*, *59*(10), 5364-5375. 28. Oshima, Y., Kamijou, A., Moritani, H., Namao, K., & Ohizumi, Y. (1993). Vitisin A and cis-vitisin A, strongly hepatotoxic plant oligostilbenes from Vitis coignetiae (Vitaceae). *The Journal of Organic Chemistry*, *58*(4), 850-853. 29. Oshima, Y., Namao, K., Kamijou, A., Matsuoka, S., Nakano, M., Terao, K., & Ohizumi, Y. (1995). Powerful hepatoprotective and hepatotoxic plant oligostilbenes, isolated from the oriental medicinal plantVitis coignetiae (Vitaceae). *Experientia*, *51*(1), 63-66. 30. Pezet, R., Perret, C., Jean-Denis, J. B., Tabacchi, R., Gindro, K., & Viret, O. (2003). δ-Viniferin, a resveratrol dehydrodimer: one of the major stilbenes synthesized by stressed grapevine leaves. *Journal of Agricultural and Food Chemistry*, *51*(18), 5488-5492. 31. Pryce, R. J., & Langcake, P. (1977). α-Viniferin: an antifungal resveratrol trimer from grapevines. *Phytochemistry*, *16*(9), 1452-1454. 32. Reniero, F., Rudolph, M., Angioni, A., Bernreuther, A., Cabras, P., & Mattivi, F. (1996). Identification of two stilbenoids from Vitis roots. 33. Shinoda, K., Takaya, Y., Ohta, T., Niwa, M., Hisamichi, K., Takeshita, M., & Oshima, Y. (1997). Vitisins D and E, novel oligostilbenes from Vitis coignetiae stem barks. *Heterocycles*, (46), 169-172. 34. Takaya, Y., Terashima, K., & Yan, K. X. (2003). (+)-Viniferol D, A new stilbenetrimer from the stem of vitis Vinifera'Kyohou'. *Heterocycles*, *60*(6), 1433-1439. 35. Waffo Teguo, P., Fauconneau, B., Deffieux, G., Huguet, F., Vercauteren, J., & Mérillon, J. M. (1998). Isolation, identification, and antioxidant activity of three stilbene glucosides newly extracted from Vitis vinifera cell cultures. *Journal of Natural Products*, *61*(5), 655-657. 36. Yan, K. X., Terashima, K., Takaya, Y., & Niwa, M. (2001). A novel oligostilbene named (+)-viniferol A from the stem of Vitis vinifera ‘Kyohou’. *Tetrahedron*, *57*(14), 2711-2715. 37. Yan, K. X., Terashima, K., Takaya, Y., & Niwa, M. (2002). Two new stilbenetetramers from the stem of Vitis vinifera ‘Kyohou’. *Tetrahedron*, *58*(34), 6931-6935. | | | | | |
